# Constipation in a Patient With Thymoma: A Diagnostic Mimic of Myasthenia Gravis

**DOI:** 10.7759/cureus.93097

**Published:** 2025-09-24

**Authors:** Nirmeen Maali, Amro Ghanim, Sathyanarayana Gowda

**Affiliations:** 1 Acute Medicine, Southend University Hospital, Mid and South Essex NHS Foundation Trust, Southend-on-Sea, GBR; 2 Emergency Medicine, Princess Alexandra Hospital (PAHT), Harlow, GBR

**Keywords:** constipation, gastrointestinal dysmotility, myasthenia gravis, paraneoplastic syndrome, seronegative myasthenia gravis, thymoma

## Abstract

Constipation is a common complaint, but may present atypically in patients with underlying malignancies. We present a 54-year-old man with metastatic thymoma who was admitted with a one-week history of constipation, abdominal discomfort, and nausea. Imaging showed proximal colonic fecal loading with sparing of the descending colon, raising suspicion of dysmotility possibly secondary to a paraneoplastic syndrome. Myasthenia gravis (MG), a known thymoma-associated disorder, was suspected. However, acetylcholine receptor and muscle-specific tyrosine kinase (MuSK) antibodies were negative, and no neuromuscular symptoms were present. The patient responded well to conservative management and was discharged. Two months later, he was readmitted with rapidly progressive bulbar and generalized weakness, severe dysphagia, diplopia, and ptosis, raising renewed concern for MG. Despite persistently negative antibodies, he improved with pyridostigmine and corticosteroids. This case illustrates the evolving nature of paraneoplastic MG, the need to consider both common and rare causes of gastrointestinal symptoms in thymoma patients, and the importance of avoiding premature diagnostic anchoring.

## Introduction

Thymoma is a rare neoplasm arising from thymic epithelial cells, accounting for approximately 20% of mediastinal tumors in adults [1]. Beyond its local effects, thymoma is strongly associated with autoimmune and paraneoplastic syndromes, most notably myasthenia gravis (MG). MG occurs in around 30-50% of patients with thymoma, while thymoma itself is identified in 10-15% of patients diagnosed with MG, underlining the close relationship between these conditions [2].

The link between thymoma and MG is thought to arise from defective thymic selection, leading to autoreactive T cells that stimulate antibody production against neuromuscular junction proteins, most commonly acetylcholine receptor (AChR) antibodies [1,3]. Although AChR antibodies are highly specific, up to 15% of generalized MG and nearly 50% of ocular MG cases may be seronegative, complicating the diagnosis [3].

Clinically, MG predominantly affects skeletal muscles, with typical manifestations including fluctuating weakness, ptosis, and diplopia [1]. However, in rare and severe cases, autonomic involvement may extend to smooth muscle, including the gastrointestinal (GI) tract [4]. Gi dysmotility, constipation, and pseudo-obstruction are therefore unusual but clinically significant in patients with thymoma and suspected MG [4,5].

We report this case to highlight the importance of broad differential diagnosis when evaluating GI symptoms in thymoma patients, to avoid premature attribution to paraneoplastic MG.

## Case presentation

A 54-year-old man with a history of recurrent thymoma presented in June 2025 with a one-week history of constipation. Despite using laxatives at home, he reported minimal bowel movement, consisting only of a single episode of watery diarrhea, and described associated abdominal discomfort, nausea, and one episode of vomiting of partially digested food. He experienced an urge to defecate but had difficulty passing stool. He continued to pass flatus until the night before admission.

On arrival to the emergency department, his vital signs were stable (blood pressure: 134/78 mmHg, pulse: 86 bpm, temperature: 36.8 °C, respiratory rate: 16/min, oxygen saturation: 97% on room air), with no postural drop. His abdomen was soft but mildly tender. Neurological examination was normal, with no cranial nerve, bulbar, respiratory, or limb weakness identified.

Past medical history included recurrent thymoma (World Health Organization classification: B1 80%, B2 20%), treated with surgical resection and adjuvant radiotherapy in 2018 (60 Gy in 30 fractions), and surgical excision of recurrent nodules involving the lung, pericardium, and diaphragm in May 2022. He also had possible sleep apnoea. A CT scan in April 2025 demonstrated progression of right-sided pleural nodules without evidence of new metastatic disease.

Initial laboratory investigations were unremarkable, with normal full blood count, renal function, liver profile, thyroid function, and inflammatory markers (C-reactive protein (CRP): 1 mg/L). Serum calcium was 1.18 mmol/L (1.12-1.32). Results are shown in Table 1.

**Table 1:** Summary of relevant laboratory investigations CRP: C-reactive protein; TSH: thyroid-stimulating hormone

Parameter	Result	Reference Range
Hemoglobin (g/L)	166	130–180
White Cell Count (×10⁹/L)	6.9	4.0–11.0
Platelets (×10⁹/L)	192	150–400
Sodium (mmol/L)	139	133–146
Potassium (mmol/L)	4.1	3.5–5.3
Urea (mmol/L)	7	2.5–7.8
Creatinine (µmol/L)	111	60–110
Calcium (mmol/L)	1.18	1.12–1.32
CRP (mg/L)	1	<5
TSH (mU/L)	2.1	0.4–4.0

**Figure 1:**
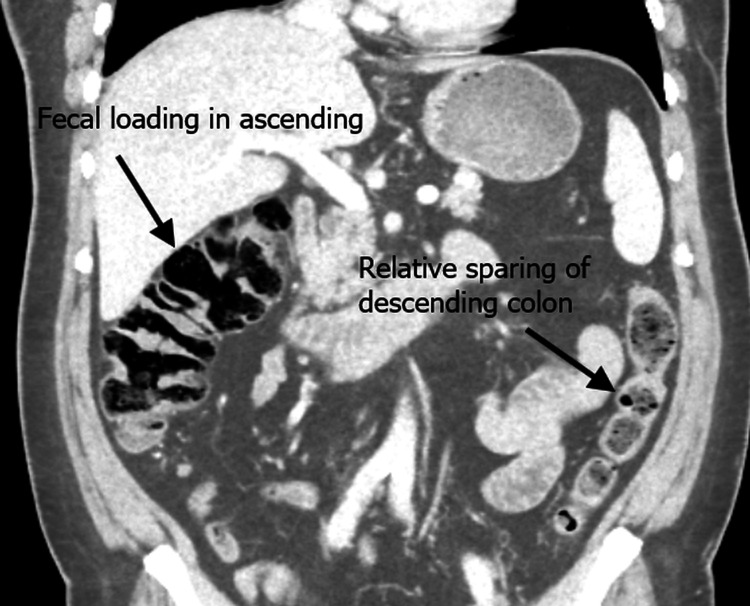
Coronal CT scan demonstrating fecal loading in the ascending colon with relative sparing of the descending colon

**Figure 2:**
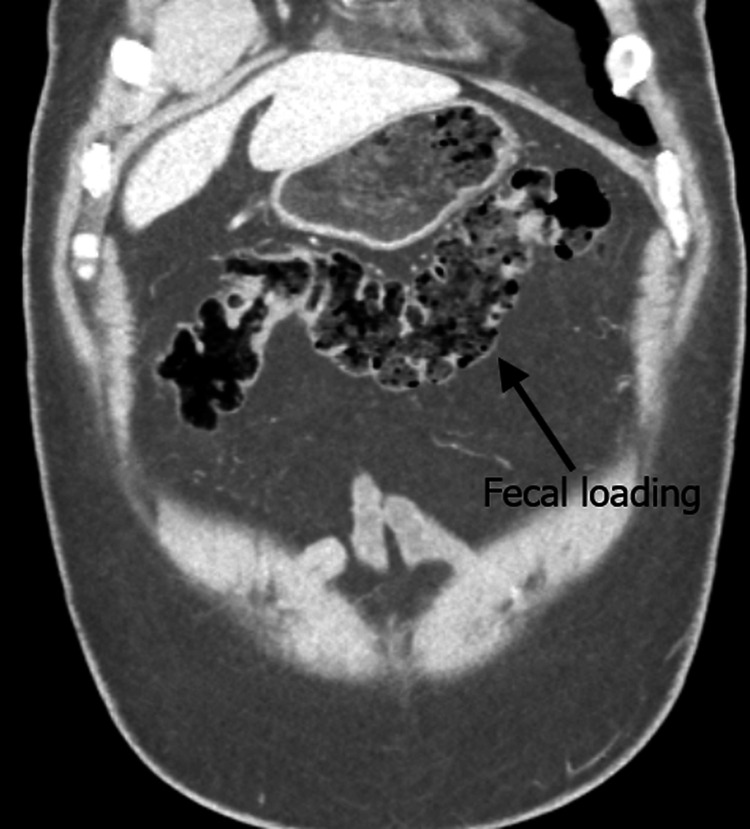
Axial CT scan demonstrating fecal loading in the transverse colon

Abdominal radiography demonstrated fecal loading throughout the colon without signs of obstruction. A contrast-enhanced CT of the abdomen and pelvis revealed diverticulosis without obstructive pathology, with marked fecal loading in the ascending and transverse colon and relative sparing of the descending colon. No mechanical cause for obstruction was identified. These findings were noted in the context of a known metastatic thymoma involving the pleura and pericardium. Figure 1 shows a coronal CT view demonstrating fecal loading in the ascending colon, and Figure 2 shows an axial CT view with fecal loading in the transverse colon.

Immunological testing showed negative anti-AChR antibody, weakly positive ANA (non-specific), and negative anti-CCP antibody. Anti-muscle-specific tyrosine kinase (MuSK) testing was pending at discharge. He was managed with enemas and laxatives, resulting in full resolution of constipation. He was discharged with outpatient follow-up arranged.

In August 2025, the patient re-presented with new and progressive neuromuscular symptoms. He reported worsening fatigue, double vision, drooping eyelids, dysphagia (progressing to inability to swallow even saliva), constipation lasting two weeks, and urinary hesitancy with post-void dribbling. He had lost four stone over two months due to poor oral intake.

Examination revealed hoarseness of voice, bilateral ptosis, diplopia, and limb weakness (upper limbs: 5/5, lower limbs: 4/5). Vital signs showed oxygen saturation of 94% on room air (NEWS 1). Neurological review confirmed bulbar and generalized weakness. CT head excluded intracranial pathology. CT thorax/neck demonstrated stable thymoma disease without new progression.

Upper GI endoscopy excluded structural dysphagia, and an NJ tube was placed. Repeat serology confirmed negative AChR and MuSK antibodies. Given the strong clinical suspicion of MG, he was started on pyridostigmine 60 mg QDS, with improvement in swallowing and strength. Speech and language therapy advised NBM and NG feeding due to dysphagia and dysphonia.

A few days later, he deteriorated with progressive weakness and desaturation, requiring up to 6 L/min O₂. Spirometry could not be performed due to weakness. Prednisolone 5 mg OD was commenced, with subsequent clinical improvement. Oxygen requirement was reduced to 1 L/min, and swallowing improved. The patient remained inpatient, awaiting EMG studies.

## Discussion

Constipation is one of the most common GI complaints encountered in clinical practice, but in oncology patients, it requires careful evaluation to exclude secondary causes. In thymoma patients, MG must be considered due to their close association [1,2], although other etiologies, such as hypothyroidism, hypercalcemia, diabetes-related dysmotility, opioid use, and functional constipation, must also be excluded. In this case, baseline labs (thyroid function, calcium, renal profile, and inflammatory markers) were all normal, and the patient was not on opioids, making these less likely.

The first admission demonstrated atypical GI symptoms without neuromuscular features. Although radiology suggested dysmotility, the absence of ocular, bulbar, or respiratory involvement and negative antibody tests supported conservative management.

The second admission highlighted the evolving nature of paraneoplastic MG, with the emergence of classical features, such as ptosis, diplopia, dysphagia, generalized weakness, and respiratory compromise. Antibodies remained negative, consistent with seronegative MG, which accounts for up to 15% of generalized cases [1,3,5]. His improvement with pyridostigmine and corticosteroids supported the diagnosis.

Compared to prior reports where GI dysmotility occurred alongside systemic MG, this patient initially presented with isolated constipation, which resolved with conservative management [4,5]. However, he subsequently re-presented with classical MG features including bulbar and respiratory involvement, demonstrating the evolving nature of thymoma-associated MG.

Therefore, this case underscores several important clinical lessons: (1) atypical symptoms in thymoma patients should prompt consideration of paraneoplastic syndromes, (2) negative antibody testing does not fully exclude MG but clinical correlation remains paramount, (3) clinicians should consider both common and rare causes of constipation in thymoma patients and avoid premature anchoring to paraneoplastic syndromes when classical features are absent, and (4) follow-up is essential, as early nonspecific presentations may evolve into overt paraneoplastic disease.

## Conclusions

In thymoma patients, GI symptoms may raise concern for paraneoplastic MG, but alternative diagnoses, such as metabolic, functional, and structural causes, must first be excluded. This case demonstrates how initially nonspecific symptoms such as constipation may precede the later emergence of classical MG features. Awareness of seronegative MG is important, but diagnosis should ultimately be guided by clinical presentation and treatment response rather than antibody status alone. Acknowledging diagnostic uncertainty and ensuring a broad differential diagnosis are crucial to avoid missed or delayed recognition of evolving paraneoplastic disease.
